# The Medico-Social Aspects of the Consumption Problem

**Published:** 1913-05-24

**Authors:** 


### THE MEDICO-SOCIAL ASPECTS OF THE CONSUMPTION PROBLEM.

### Improvising Home Accommodation.

The consumptive is apt to presuppose that unless he obtains sanatorium treatment at one of those well-equipped hotels for the sick poor which Mr. Lloyd George is never tired of expiating upon, his case is a miserable one and his chance of ultimate recovery almost *nil*. This is a great mistake. Even when all the arrangements have been perfected and we have sanatoria scattered all over the country, it is likely that there will not be enough beds for all diseased persons, while in addition there will be a class of consumptives who for various reasons will not be able to take advantage of the facilities offered by the State sanatoria. These patients can do much for themselves. Indeed there is perhaps no disease in which the energy and self-help of the patient are of so much importance as in consumption, since without them it is difficult, if not impossible, to hope for any good results from the best methods of treatment. If a consumptive takes an interest in his own case; if he be made to recognise the possibilities in the way of cure, and the danger of delay both in enhancing the degree of severity of his own case and in increasing his potentiality as a factor of spreading disease, much will have been done to make him at the same time a joyous example of the benefits that may result from such self-help.

Home treatment holds out chances for those consumptives who cannot afford to go to a sanatorium, and it shows also how much individual effort and self-help may do to keep the disease in check, prevent the further spread of it, and put the patient in a position to be economically useful without removing from his business environment. The essentials of home treatment are similar to those of sanatorium treatment, though the details are not. In a sanatorium the whole establishment has been built and arranged to suit the patients; at home the consumptive must adapt himself to the conditions and circumstances of his home as he finds it and must not expect that everything will be made subservient to his interests. This is especially the case in poor homes where the ordinary conveniences of nursing are lacking. For all this, a great deal can be done to make up for the want of these facilities, and we will here try to give a short outline of the manner in which a commencement may be made.

### How to Construct a Sleeping Shelter.

The first thing the consumptive will have to obtain is a private sleeping-room which he uses alone. Taking it for granted that he avails himself during the daytime of every opportunity for obtaining fresh air by working in a shop with open windows and thorough ventilation, by taking his midday meal in the park or somewhere in the open, and by walking home or riding outside on 'bus and tram and so giving himself every chance, consistent with the avoidance of undue exposure, to fill his lungs with fresh air—it yet remains for him to see that he gets equal opportunities at night. If he shares a room, apart from the ordinary hygienic considerations which condemn such partnership between a healthy individual and a consumptive, there is always the risk that his room-mate may object to sleeping with open windows, for the principles of free ventilation are not yet grasped by the community as a whole. He should, therefore, try and get a sleeping shelter. This does not mean an expensive structure such as is catalogued by the dealers, prices of which range from six to fourteen guineas, but is something that may be built by the consumptive patient himself out of such material or remnant of materials as he can purchase or beg for a few shillings. A bamboo framework with walls of lathing or thin deal or bass wood is all that is required, provided the roof is made fairly strong, sloping, and waterproof. The cheapest shelters are those made of Japanese matting, but they do not serve well in this climate except in summer. The size and shape of the shelter will depend on the floor space available and the site, and also on the workman's ingenuity and the extra expenditure he can afford. Ordinarily it should be large enough to allow of a single bedstead to be placed in it, with two feet room all round the bed. Its door and windows should be made of simple matting, so that they can be pushed close when it rains but turned sideways or kept rolled up when it is fine weather. The bedstead should be screwed or clamped to the floor, and the bedding removed during the day for airing. The shelter itself should be securely fixed to its site so as to prevent disturbance by wind or vibrations.

Such a shelter can be erected anywhere where the patient can obtain permission to establish it, provided of course that the selected site is really suitable for open-air treatment shelters. A back garden or area or a flat roof does excellently. Many old houses have flat back leadings abutting from the first floor and overlooking area or backyard; these are suitable sites for such a shelter and are usually vacant, since the tenants of the first floor rarely use them; a very low rent is charged by some landlords for the use of these leadings by tenants on the higher or lower floor who wish to use them, or an arrangement is made with the first-floor tenants themselves. The patient should exercise his ingenuity not only in building the shelter but in finding a site whereupon to plant it when it has been made. In thickly crowded industrial quarters it may be very difficult to find an ideal shelter site; in that case the second best site must be chosen, care being taken that three essentials are kept in view—viz. that the site be really an open-air site, and not so enclosed by surrounding buildings that it is shut off from light and air; that it be moderately private and free from disturbing noises; and that it be well away from anything that may be considered a nuisance such as a mews or stables, fowl-house or piggery. It is sometimes advisable for the patient to go out of his industrial district in looking for a site. For instance, working-men in the East End of London will find good sites in the less crowded areas towards Plaistow and along the riverside, where a moored barge sometimes serves admirably for a time. In these cases, of course, the patient must arrange for his meals, washing, care of bed-clothes, etc., somewhere in the neighbourhood; but the little extra trouble is well repaid by the resultant satisfaction at the results obtained through the possession of a good site.

### The Selection of a Bedroom.

Where the building of a shelter is, for various reasons, out of the question (the main reason nearly always being the difficulty of providing an adequate site) the patient may turn his mind to converting his bedroom into a shelter after the type of the sanatorium room. When this is done it is, of course, imperative that he should be the sole occupant of the room. He should choose, if possible, a room on the top storey of the house, and one that is moderately small and with a moderately low ceiling, though a ceiling below ten feet is not advisable. He should, in particular, see that the windows open well and that the window space is adequate. If not, he must endeavour in some way to enlarge them. It is preferable to do this by lengthening them, since this method gives a better air current when the window is open, while the rearrangement does not interfere with the wall so much and is therefore easy for the workmen to carry out. Structural alteration may in many cases be done by the patient himself if he is a handy man, of course with the landlord's approval and consent; in that case the attic room may be made into an ideal bedroom for a consumptive patient, with large air supply and free through ventilation, so that all the conditions for open-air treatment are obtained.

If it be possible to get a hold for tent-pegs, expenses may be reduced very much. We have known a roomy weatherproof tent to be bought second-hand for 25s. A wooden tent-bottom can be made by anyone out of soap-boxes, and all that is needed else is the bed. The bottom of the tent canvas is "brailed up" at night to a height of two feet, thus securing both privacy and ventilation.

In a future article we intend to give some illustrations showing various ways in which such rooms can be adapted and of shelters constructed by working-men patients for their own use. We lay some stress upon these points since it seems to us that the public does not fully recognise, as yet, how comparatively easy and simple it is to obtain conditions of ventilation, approaching those that obtain in well-equipped sanatoria at home, and with a fraction of the cost expended on institutional refinements. The results of treatment carried out at home under these conditions may not be comparable with those obtained at the sanatoria since the treatment is not so thorough and the patient, in the majority of cases, carries on his ordinary work while he is undergoing treatment and is exposed to the evils of his workshop or industry. But they are good in so far as they ameliorate the conditions of disease; give the patient a better chance to recover; maintain his economic value; and by isolating him prevent him from being a danger to the rest of his fellow-citizens. For that reason the value of home treatment should be impressed on the consumptive patient who for various reasons cannot afford to go to a sanatorium.

